# Performance of a multiplexed dual analyte immunoassay for the early detection of non-small cell lung cancer

**DOI:** 10.1186/s12967-015-0419-y

**Published:** 2015-02-12

**Authors:** Victoria Doseeva, Tracey Colpitts, Grace Gao, Juliana Woodcock, Vladimir Knezevic

**Affiliations:** 20/20 GeneSystems, 9430 Key West Avenue, Rockville, MD 20850 USA; Abbott Molecular Inc, 1300 E Touhy Avenue, Des Plaines, IL 60018 USA

**Keywords:** Lung cancer (LC), Biomarkers, Early detection, Tumor proteins, Autoantibodies, Blood test

## Abstract

**Objectives:**

“PAULA’s” test (*P*rotein *A*ssays *U*tilizing *L*ung cancer *A*nalytes) is a novel multiplex immunoassay blood test that incorporates both tumor antigens and autoantibodies to determine the risk that lung cancer (LC) is present in individuals from a high-risk population. The test’s performance characteristics were evaluated in a study using 380 retrospective clinical serum samples.

**Methods:**

PAULA’s test is performed on the Luminex xMAP technology platform, and detects a panel of 3 tumor antigens (CEA, CA-125, and CYFRA 21–1) and 1 autoantibody marker (NY-ESO-1). A training set (*n* = 230) consisting of 115 confirmed diagnoses of non-small cell lung carcinoma (NSCLC) cases and 115 age- and smoking history-matched controls was used to develop the LC predictive model. Data from an independent matched validation set (*n* = 150) was then used to evaluate the model developed, and determine the ability of the test to distinguish NSCLC cases from controls.

**Results:**

The 4-biomarker panel was able to discriminate NSCLC cases from controls with 74% sensitivity, 80% specificity, and 0.81 AUC in the training set and with 77% sensitivity, 80% specificity, and 0.85 AUC in the independent validation set. The use of NY-ESO-1 autoantibodies substantially increased the overall sensitivity of NSCLC detection as compared to the 3 tumor markers alone. Overall, the multiplexed 4-biomarker panel assay demonstrated comparable performance to a previously employed 8-biomarker non-multiplexed assay.

**Conclusions:**

These studies confirm the value of using a mixed panel of tumor antigens and autoantibodies in the early detection of NSCLC in high-risk individuals. The results demonstrate that the performance of PAULA’s test makes it suitable for use as an aid to determine which high-risk patients need to be directed to appropriate noninvasive diagnostic follow-up testing, especially low-dose CT (LDCT).

## Background

In the United States, LC is the third most commonly diagnosed cancer, with approximately 224,210 new cases expected in 2014 [1]. Moreover, LC accounts for more combined cancer-related deaths in men and women than any other cancer (approximately 27% of all cancer-related deaths in a recent study) [1]. Cigarette smoking remains the single most important determinant of LC susceptibility and is responsible for 87% of LC deaths among men and 70% of LC deaths among women [2]. The relative risk of death from LC is about 26 times higher in female smokers and 25 times higher in male smokers compared to lifelong nonsmokers [3]. Risk increases with quantity and duration of cigarette consumption [4].

While the overall survival rate for LC is 15%, a survival rate of 70–80% can be achieved when detected early in individuals with localized cancer [5]. Yet only 15% of LCs are diagnosed at this early stage [1]. These statistics imply a compelling unmet need for new noninvasive tests for the early detection of LC. CT is the most commonly used method for regular screening for the early detection of LC in clinical settings [6]. Despite its relatively good sensitivity for LC detection (93.8%) [7], CT has many drawbacks that suggest its applicability will be limited as a stand-alone detection methodology. These problems include a high false-positive rate (including the inability to unambiguously distinguish benign nodules that can involve expensive invasive follow-up procedures); the high cost of testing; and the danger of cumulative diagnostic radiation exposure with repeated testing [8-10]. Another major drawback of low-dose computed tomography (LDCT) scanning is over-diagnosis, which has been estimated to be more than 18.5% for all LCs [11].

For these reasons, the development and validation of a robust and cost-effective early-stage blood test to complement imaging-based diagnosis is essential. The literature reports a variety of approaches that have been pursued for nearly 2 decades to reach this end, including (1) proteomics-based methods, (2) characterization of genomic expression, (3) monitoring gene methylation, (4) detection of mitochondrial DNA mutations or chromosomal abnormalities, and (5) the detection of such blood-based biomarkers as tumor-derived proteins, microRNA, or autoantibodies arising from a patient’s immune response to tumor cells [12-14]. A number of recent review papers indicate blood-based biomarker detection is the most highly developed of these approaches and support the concept that these biomarkers can be used to detect early stage NSCLC and complement CT imaging [15-18].

Many investigators have demonstrated that tumor antigens are present in the serum or plasma of patients with LC at all stages. Technologies used to detect and quantify tumor protein expression have varied from traditional ELISA methodology [19-21], to electrochemiluminescence immunoassay [22,23], modified aptamers (so-called slow off-rate modified aptamers [SOMAmers]) [24,25], MALDI MS proteomic analysis [26,27], and multiplex Luminex assays [28,29]. Among the most promising approaches is an ELISA-based test [20] in which 4 serum proteins (CEA, RBP, 1-antitrypsin, and SCC) were used. In a validation set consisting of 97 samples (49 cancers and 48 controls), this panel demonstrated the ability to distinguish LC with 77.8% sensitivity and 75.4% specificity from matched control patients.

In 2010, Ostroff et al. [24] reported results from a proprietary proteomic assay based on SOMAmers, in which a 12 protein NSCLC proteomic signature in serum was identified. This 12-protein panel (cadherin-1, CD30 ligand, endostatin, HSP90α, LRIG3, MIP-4, pleiotrophin, PRKCI, RGM-C, SCF-sR, sL-selectin, and YES) discriminated NSCLC from controls with 89% sensitivity and 83% specificity in a blinded validation study (341 samples).

Results from another multiplex Luminex-based assay for the detection of early stage NSCLC using protein biomarkers was reported by Bigbee et al. in 2012 [28]. In this study, the authors evaluated the combined effect of 10 protein biomarkers (prolactin, transthyretin, thrombospondin-1, E-selectin, C-C motif chemokine 5, macrophage migration inhibitory factor, plasminogen activator inhibitor, receptor tyrosine-protein kinase, CYFRA 21–1, and serum amyloid A) for the ability to distinguish LC from controls. The assay’s discriminative performance was demonstrated in a blinded verification set consisting of 60 samples (73.3% sensitivity at 93.3% specificity). Based on the observed panel performance, the authors concluded that these biomarkers could potentially improve interpretation of CT images in the setting of suspicious pulmonary nodules.

Several publications have appeared in recent years documenting incorporation of protein biomarkers into CT imaging models [27,30-32]. Yildiz et al. [26] identified a serum proteomic signature to distinguish LC cases from matched controls with MALDI MS technology. Their MALDI MS proteomic analysis reached an overall accuracy of 72.6 %, a sensitivity of 58%, and a specificity of 85.7% in a blinded test set. A later study [27] demonstrated that this proteomic signature was able to discriminate LC from benign indeterminate lung nodules. These studies were among the first to demonstrate the additive value of protein biomarkers to pulmonary nodules imaging results.

In addition to detecting tumor antigens directly, detecting autoantibodies to tumor antigens has also been extensively investigated as an alternative approach for the early detection of LC [33-36]. Use of autoantibody detection is very promising, since autoantibodies have been shown to be present and detectable in patient blood as much as 5 years before some patients present with symptoms [37-39]. One of the first studies on the use of autoantibodies for early detection of LC was described in publications by Hanash et al. [39-41]. The authors identified 3 autoantibodies (annexin 1, LAMR 1, and 14-3-3 theta) that are found prior to diagnosis in about 30% of the LC patients [39]. This autoantibody panel yielded 55% sensitivity at 95% specificity and 0.838 AUC in discriminating LC at the preclinical stage from matched controls. Zhong et al. [37] reported a panel of 5 autoantibodies that yielded 91.3% sensitivity and 91.3% specificity in the prediction of NSCLC cases, although with a limited sample set, and not including patients with nonmalignant pulmonary nodules. Farlow et al. [42] identified a 6-autoantibody panel consisting of IMPDH, phosphoglycerate mutase, ubiquillin, annexin I, annexin II, and HSP70-9B that can detect NSCLC among several patient cohorts including benign lung diseases (chronic obstructive pulmonary disease [COPD]/asthma) and benign pulmonary nodules. Their Luminex-based assay showed impressive performance characteristics: an AUC of 0.964, a sensitivity of 94.8%, and a specificity of 91.1%, which compares quite favorably to previously published autoantibody panels.

Several publications from Oncimmune Corp. (Nottingham, UK) provided further evidence supporting the use of autoantibodies for early detection of NSCLC [43-46]. Murray et al. [43] first described a laboratory-developed test for the early detection of LC based on the presence of 6 autoantibodies (CAGE, GBU4-5, HuD, MAGE A4, NY-ESO-1, p53, and SOX) in the serum of heavy smokers. The clinical performance of this test has been confirmed in research studies and in clinical settings yielding a specificity of 82-91% and a sensitivity of 37-41% [44-46]. Overall, the development of these tests suggests that autoantibodies can be used as effective biomarkers in the detection of NSCLC. However, the data also indicate that to achieve significant sensitivity and thus broader clinical acceptance, either a larger autoantibody panel or a panel including other biomarkers will be required.

As shown, there is a rapidly growing literature on both tumor proteins and autoantibodies as biomarkers for the early detection of LC. Tumor protein biomarkers and autoantibody biomarkers are measurable in the blood of individuals with various forms of solid tumors and may be detected before the appearance of clinical symptoms in late-stage disease [17,37-39]. Interestingly, no publications can be found in the literature that describe diagnostic assays using mixed panels of tumor markers and autoantibodies. The use of such a mixed panel, if properly selected, could have the benefit of combining the superior sensitivity of protein biomarkers with the high specificities observed in some of the autoantibody studies.

The objective of this study was to evaluate the performance of our dual analyte PAULA’s test with early stages of NSCLC in order to confirm the ability of our mixed biomarker panel to identify early tumors in high-risk patients where disease is curable. One goal was to evaluate whether a combination of tumor protein and autoantibody detection will raise the performance level of a diagnostic panel. Here we report a new retrospective study of PAULA’s test using a total of 380 patients from 3 cohorts consisting of 190 confirmed diagnoses of LC and 190 age- and smoking history-matched controls. Overall, the results show that our 4-biomarker panel was able to discriminate early-stage NSCLC cases from controls with 74% sensitivity, 80% specificity, and 0.81 AUC in the training set (*n* = 230) and with 77% sensitivity, 80% specificity, and 0.85 AUC in the independent validation set (*n* = 150). We believe that the performance of PAULA’s test, as well as its simple and easy-to-use format, makes it a viable candidate assay for use as an aid to determine which high-risk patients need to be directed to appropriate diagnostic CT scanning, due to its reduced false-positive rate, low cost, and reduced risk (with respect to radiation exposure), as compared to CT screening.

## Materials and methods

### Serum samples

All the cancer and normal control samples used in this study were IRB-approved, consented serum samples that were purchased from Clinical Research Center of Cape Cod, Inc. (Cape Cod, MA) and Asterand (Detroit, MI). Lung cancer serum samples were collected at 3 different United States sites and in 3 European countries (Ukraine, Russia, and Romania) to control for pre-analytical variability. All of the lung cancer samples were collected at physicians’ offices or hospitals. Many of the control samples were collected at Cape Cod Clinical Research center. Not all of the cases and controls from the US were matched on a site-specific basis, which may be a source of pre-analytic variability.

All NSCLC serum samples were within the PAULA’s test acceptance criteria: patients 50 years of age or older who were current or former smokers with a smoking history of greater than 20 packs per year and less than 15 years of smoking cessation. Diagnosis confirmation for the NSCLC cohort was obtained from surgical pathology reports.

The control cohort was selected on the basis of similar demographic characteristics (with respect to age and gender) to the NSCLC cohort. The control serums were from healthy volunteers matched for smoking history of greater than 20 packs per year. The control group had no evidence of any current or prior cancer.

Additional control serum samples with benign lung diseases and other cancers were provided by Dr. S. Radulovich and were collected in her Clinical Laboratory Improvement Amendments (CLIA) laboratory (Bel Air, MD) in accordance with IRB protocol and CLIA regulations. This cohort comprised 81 serum samples from patients with nonmalignant diseases (e.g., COPD, asthma, bronchitis, pulmonary fibrosis, pneumonia, emphysema, chronic inflammation, etc.) and cancers other than NSCLC (e.g. myeloma, breast and colon cancers).

### Multiplex assay

Multiplexed serum immunoassays were performed using the xMAP technology platform (Luminex Corporation, Austin, TX). Commercially available reagents from Luminex, and Millipore, Inc. as well as reagents developed in house (20/20 Gene Systems, MD) were used. MagPlex-carboxylated polystyrene beads were purchased from Luminex. MagPlex beads with conjugated antibodies against carbohydrate antigen 125 (CA125) and CYFRA 21–1, as well as detection antibodies for CEA, CA125, and CYFRA 21–1 proteins were purchased from Millipore, Inc. The capture antibody for CEA was purchased from Abcam (Cambridge, MA).

#### Luminex bead coupling

Covalent coupling of the CEA capture antibodies to the microspheres was done by the procedures recommended by Luminex Corp. In short, 5x10^5^ microspheres were dispersed by vortexing, washed with 100 μL of deionized water, and resuspended in a microtiter tube with 80 μL activation buffer (100 mM monobasic sodium phosphate, pH 6.2, Sigma). To activate the microspheres 10 μL of 50 mg/mL sulfo-NHS (EZ-Link sulfo-N-hydroxysuccinimide ester, Thermo Scientific) and 10 uL of 50 mg/mL EDC (1-ethyl-3-(3-dimethylaminopropyl) carbodiimide hydrochloride, Thermo Scientific) were added to the beads. The bead suspension was incubated in the dark for 20 min at room temperature. Following incubation the activated beads were washed twice in coupling buffer (0.05 M MES, pH 5.0, Sigma) and resuspended in 100 μL of the same buffer. 5 μg of anti-CEA antibody (Abcam) was added to the mixture and the reaction was incubated for 2 hours at room temperature in the dark with continuous shaking. The microspheres were then washed twice with PBS containing 0.1% of BSA, 0.02% Tween-20, and 0.05% sodium azide, pH 7.4, counted using the MAGPIX instrument, and stored in the dark at 4°C.

Recombinant NY-ESO-1 protein was obtained from Thermo Fisher Scientific, Inc. (Rockford, IL). It was conjugated with MagPlex microspheres (Luminex Corp.) using a slightly different bead coupling protocol than described above. 2.5 μg of recombinant NY-ESO-1 protein was added to 1.25x10^6^ microspheres in 200 μL 0.05 M MES buffer, pH 5 (Sigma) and incubated for 2 hours at room temperature in the dark. The mixture was subsequently combined with 20 μL of 1 mg/mL EDC. Following overnight incubation at room temperature in the dark a total of 4 washes with 1 mL of PBS/1% BSA/0.2% Tween-20/0.04% sodium azide were performed. The conjugated beads were resuspended in 500 μL of blocking/storage buffer (PBS-TBN contains 0.1% of bovine serum albumin, 0.02% tween-20, and 0.05% sodium azide, pH 7.4) and stored at 2–8°C in the dark.

#### Tumor marker multiplexed serum immunoassay

Multiplexed serum immunoassays were performed using the xMAP technology platform (Luminex Corp.). Samples were assayed in triplicate. Each serum sample was incubated overnight at 4°C with the mixture of approximately 800 capture antibody-conjugated magnetic microspheres per protein analyte per well in 96-well microtiter plate (Millipore). Following washing with PBST buffer, the antigen-antibody complex was incubated with the biotin-conjugated detection antibodies (Millipore) for 1 hour at room temperature with constant agitation. Finally, the complex was incubated with streptavidin-phycoerythrin for 30 minutes in the dark with agitation. The resulting bead complex was again washed 3 times, resuspended in Drive Fluid (Luminex reading buffer), and analyzed using the MAGPIX instrument.

For each analyte, median fluorescence intensity (MFI) values were calculated using Luminex xPONENT software. All biomarker protein concentrations were calculated using a 5-parametric curve fit as part of the xPONENT software. The calculated protein concentration values were used for the subsequent analysis.

#### Autoantibodies serum immunoassay

Briefly, each serum sample was incubated for 1 hour with approximately 1,000 NY-ESO-1-conjugated magnetic microspheres per well in 96-well microplate. The plates were washed 3 times with PBS-T wash buffer (0.01 M phosphate buffered saline with 0.05% Tween-20, pH7.4), then the immobilized autoantibodies-NY-ESO-1 protein complexes were incubated with goat anti-human IgG conjugated to R-Phycoerythrin for 30 min at room temperature on a plate shaker. Following 3 washes with PBN-T buffer (phosphate-buffered saline with 0.2% Tween-20, pH 7.4), complexes were resuspended in the same buffer and run on the MAGPIX system.

In the absence of available autoantibody concentration standards, background subtracted MFI values were used for the subsequent analysis.

#### Inter- and intra-assay precision

Intra-assay precision was calculated as the average coefficient of variation (CV) between triplicates. MFI signals for each biomarker were measured in triplicate for 20 samples. The % CV for each sample was calculated between the triplicates. The average of the individual CVs was reported as the intra-assay CV. The intra-assay variability for individual biomarkers was in the range of 3.1% to 8.1%.

The inter-assay precision was calculated from the mean values for the high and low controls on each assay plate. High and low controls for each biomarker were run in triplicate on 4 different days to monitor day-to-day variation. The plate means for high and low controls were calculated and then used to calculate the overall mean, standard deviation, and overall % CV. The average of the high and low % CV was reported as the inter-assay CV. Inter-assay precision for individual biomarkers was in the range of 2.6% to 14.1%. Thus, inter- and intra-assay reproducibility meets precision industry standards for these immunoassays.

### Statistical methods

Logistic regression was used to develop a composite panel of 4 biomarkers to distinguish between matched LC cases and controls for a training (*n* = 230) and matched validation (*n* = 150) set. The logistic regression models (PROC LOGISTIC, SAS; Cary, NC) were fitted by comparing biomarker levels (CEA, CA125, CYFRA 21–1 and NY-ESO-1) in LC cases and controls. The 4 biomarkers were tested separately and a composite score for the panel was determined using 3 approaches: (1) multiple of median (MoM) in which the data from each biomarker was converted to a multiple of a population median value by dividing by the median value of the control group; (2) multiple logistic regression (MLR) using the data from the biomarker panel and; (3) training set weightings from a multiple logistic regression of the z-transformed data from each biomarker predicting cancer versus control outcome.

The area under the receiver operator characteristic (ROC) curve (AUC) analysis was used to compare the diagnostic power of each approach. To determine sample size, an AUC with a null hypothesis (Ho) set at 0.5 was used as the benchmark. A statistical power analysis found that a sample size of 150 (75 control and 75 cases) would provide 90% power to detect an AUC of 0.7, the lowest AUC considered of clinical significance, from the null AUC of 0.5 [47]. The cohort was used to create a training data set and a matched validation data set. A sample size of 230 (115 controls and 115 cases) was used in the training data set and 150 (75 controls and 75 cases) was used in the validation set. The training set was used to develop the LC predictive model and the validation set was used to test the model developed. An AUC analysis was used to determine sensitivity and cutoff point value at 80% specificity.

### Biomarker panel selection

The work presented here is based on preliminary studies performed at Abbott Molecular, Inc, in which a large number of biomarkers were employed to screen for a wide array of lung cancer types. The primary conclusion provided by this initial work is that, to date, even assays that detect panels of several tumor protein markers do not produce sufficient sensitivity for early-stage disease detection. However, the addition of autoantibody detection to the assay increases the performance levels of a biomarker panel for early detection due to the fact that autoantibodies develop early in the oncogenesis process and are thus stage independent, unlike tumor protein biomarkers [37-39]. Thus, even at early stages of cancer, when tumors are small and there is a lower concentration of circulating biomarkers, the production of antibodies as part of the body’s response to the presence of cancerous cells is robust and amplified.

The primary approach for selecting cancer autoantibodies was via protein array technology, which utilizes microarrays of large numbers of full-length proteins. Researchers from Abbott Molecular, Inc. used the ProtoArray protein microarray from Invitrogen for discovery of autoantibodies for early detection of LC. Invitrogen’s ProtoArray (5000 proteins) was screened with sera from NSCLC patients, individuals with benign lung disease and apparently healthy controls. Autoantibodies were identified that discriminated cancer from benign and normal individuals. The criteria used for selection was that the individual autoantibodies must have complementary performance. That is, a high percentage of the cancer samples tested were detected with at least one of the autoantibodies in the selected autoantibody array. Multivariate analyses (decision tree (CART) and principal component analysis) were used to confirm the complementarity of the autoantibodies selected. This screening effort identified 4 auto-antigens that help discriminate cancer from benign lung disease and normal individuals. Two of them, MAPKAP3 and Cyclin E2, showed the best discrimination. Other antigens known to elicit autoimmune responses in cancer patients, such as p53 and NY-ESO-1, were also included in the study.

This research led to the development of a panel of 8 biomarkers capable of early detection of LC, which included tumor protein markers (CA125, CEA, CYFRA 21–1, and Pro-GRP) and 4 autoantibodies (MAPKAP3, Cyclin E2, p53, and NY-ESO-1) [48].

Markers were carefully chosen to complement each other. All tumor biomarkers (CA125, CEA, CYFRA 21–1, and Pro-GRP) from the final panel have been extensively studied and validated by others and are currently in clinical use for monitoring of other cancers. CA125 and CEA were measured on the Architect instrument, CYFRA 21–1 on the Elecsys 2010 system, and Pro-GRP on a commercially available ELISA kit (Advanced Life Science Institute Inc., Japan). Individual assays for the 4 identified autoantibodies were developed on Luminex platform.

Eight markers have subsequently demonstrated high levels of performance in multiple sample sets from independent centers. During this discovery and development period, a population of 782 people including 264 NSCLC cases representing all stages of disease, 270 biopsy-confirmed benign lung diseases, and 242 healthy smoker controls matched for gender, age, and smoking history, were studied. These serum samples were divided into 3 independent cohorts—1 for the development of marker panels, and the other 2 (a training set and a test set) for validation of those panels. Of these samples, a total of 245 met PAULA’s acceptance criteria (127 NSCLC cases and 118 controls). Early-stage NSCLC was distinguished from controls (e.g., benign lung disease and normal) with an AUC of 0.84, and 72% sensitivity at 80% specificity.

Among the noncancerous patients, the specificity of the 8-marker test was not greatly affected by either smoking status or the presence of noncancerous lung diseases (including asthma, COPD, emphysema, fibrosis, and pneumonia), all of which yielded scores in the same proportions as the overall normal population. 145 samples from 3 other high incidence cancer types (prostate, breast, and colorectal) were tested by Abbott group to ascertain the specificity of the test. Analysis of this “other cancer” group (that was left out of the control population for determination of the AUC and cutoff) demonstrated that these other cancers yielded a higher score more often than noncancerous conditions, yielding a specificity of 66% and sensitivity of 34%.

There were deficiencies with the 8-marker method that precluded it from being ready for clinical use. The most intractable problem was that the panel was run on several platforms with different detection technologies, making it impractical for commercial use. We also found that by removing 3 autoantibodies and the Pro-GRP protein tumor biomarker, leaving a 4-biomarker panel (CA125, CEA, CYFRA 21–1, and NY-ESO-1), assay performance was maintained at levels similar to the 8-marker panel, but with reduced labor and material costs. This reduced panel contains a set of biomarkers that narrows the potential application of the test to NSCLC at the expense of SCLC detection. This was considered acceptable since NSCLC is a particularly common and clinically relevant type, accounting for 86% of lung cancers [1].

## Results

### Study design

In this study, the objective was to evaluate the diagnostic accuracy of PAULA’s test in detecting early stages of NSCLC using our current 4-biomarker panel. The goal was to identify early tumors in high-risk patients (smokers or former smokers) where disease is curable.

Two independent studies were performed. Each of the studies investigated serum sample types with known diagnoses and outcomes. Results from the first study (training set) were used to develop a classification method for the samples that was then applied to the second study (validation set).

The training set consisted of 230 samples and included 115 histopathologically confirmed early (stage I and II) NSCLC cases (96.5%) and 3.5% stage III cancers (Table 1). The LCs consisted of 38% squamous cell carcinoma, 36% adenocarcinoma, 17% large cell carcinoma including neuroendocrine carcinoma (LCNEC), 7% bronchioloalveolar carcinoma, and 2% unidentified LC. The control cohort included 115 serum samples from healthy risk-matched volunteers (Table 1).

**Table 1:** **Clinical and demographic characteristics of the NSCLC cases and controls used in the training set and validation set**

**Training set**			**Validation set**		
**Histologic subtype**	**Number**		**Histologic subtype**	**Number**	
Adenocarcinoma	41		Adenocarcinoma	32	
Bronchioloalveolar carcinoma	7		Bronchioloalveolar Carcinoma	21	
Squamous cell carcinoma	45		Squamous Cell Carcinoma	15	
Large cell carcinoma	3		Carcinoma, NOS	4	
Neuroendocrine	16		Neuroendocrine	1	
Other	3		Other	2	
**Stage**			**Stage**		
Stage I	88		Stage I	23	
Stage II	23		Stage II	26	
Stage III	4		Stage III	15	
Stage IV	0		Stage IV	10	
Total	115		Total	75	
**Gender**	**Control**	**Cases**	**Gender**	**Control**	**Cases**
Females	48	49	Females	29	48
Males	67	66	Males	46	27
Total	115	115	Total	75	75
**Age**	**Control**	**Cases**	**Age**	**Control**	**Cases**
Median	64	64	Median	68	69
Range	50-97	50-88	Range	52-98	50-99
**Smoking history**	**Control**	**Cases**	**Smoking history**	**Control**	**Cases**
Packs/year	>20	>20	Packs/year	>20	>20

The validation set consisted of 150 samples and included 75 NSCLC patients from the high-risk population and 75 normal controls. 50 NSCLC cases were histopathologically confirmed as early stages I or II (67%) and 25 cases (33%) were stages III and IV (Table 1). Similar to the training set, this validation set included a range of LC histologies: 32 adenocarcinomas, 21 bronchioloalveolar carcinomas, 4 carcinomas not otherwise specified (NOS), 1 large cell neuroendocrine carcinoma, and 2 unidentified LCs. The clinical and demographic characteristics of the cases and control patients comprising this set are summarized in Table 1.

### Training set

**Figure 1:**
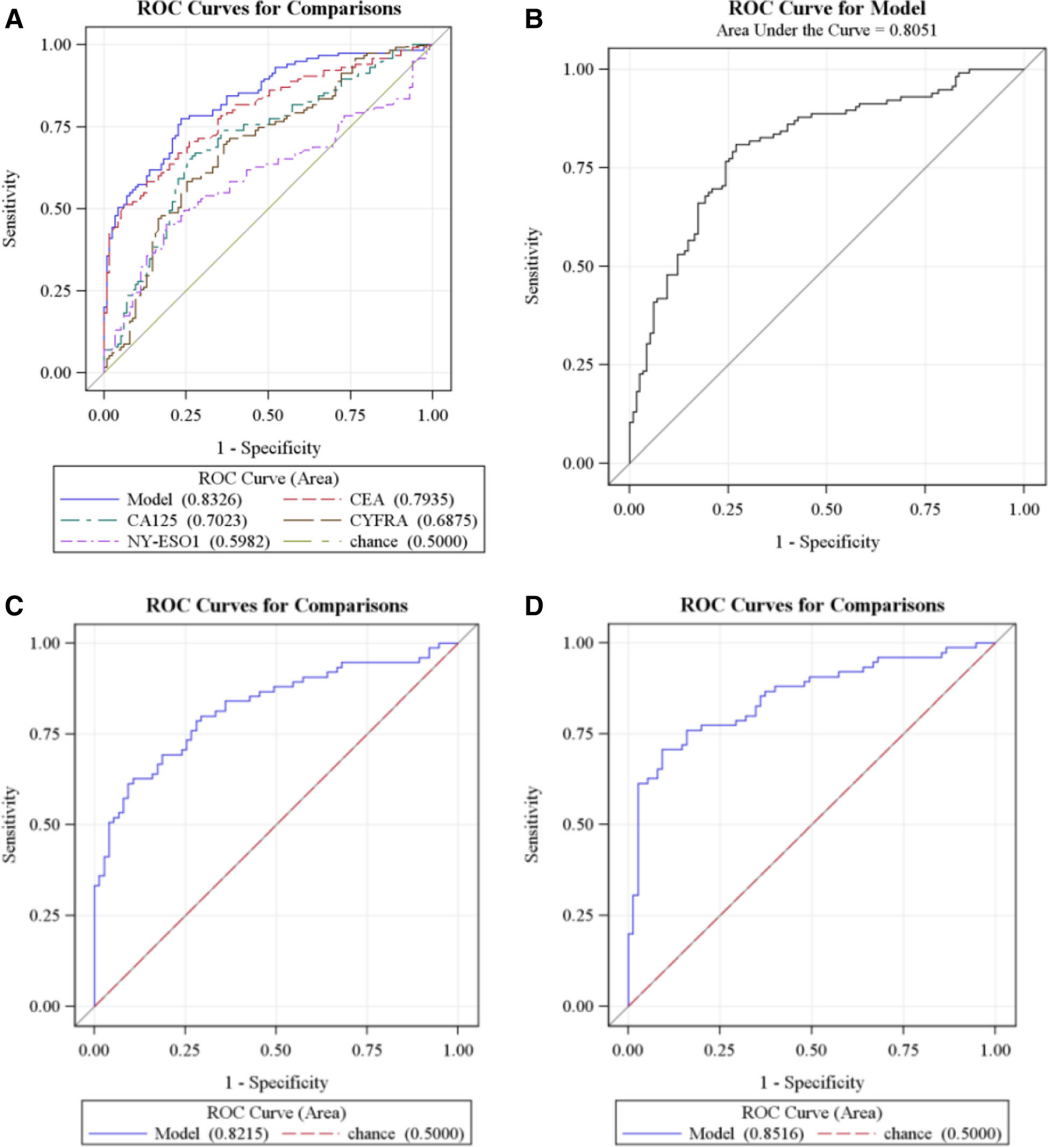
**ROC curve analyses of training data set (** ***n*** **= 230) (A and B) and validation set (** ***n*** **= 150) (C and D).** Panel **A** shows ROC curves for prediction of LC using multiple logistic regression weightings for each individual biomarker and a model combining all 4 biomarkers. The AUC values are 0.60, 0.79, 0.70, 0.69, and 0.83 for NY-ESO-1, CEA, CA125, CYFRA 21–1, and the combined panel, respectively; Panel **B** is a ROC curve generated from the 4-biomarker panel using the MoM scoring method (AUC value = 0.81); Panel **C** shows a ROC curve of the validation set for prediction of LC using the MLR method (AUC = 0.82), and Panel **D** shows the same data using MoM transformation (AUC = 0.85).

ROC curves from a logistic regression analysis were used to evaluate the capability of each of the 4 biomarkers to discriminate between LC cases and controls. The individual biomarkers exhibited AUC values in the range of 0.60–0.79 (Table 2; Figure 1A), where the highest AUC from an individual biomarker was found for CEA (0.79; Table 2; Figure 1A). The combined panel of 4 biomarkers (NY-ESO-1, CEA, CA125, and CYFRA 21–1) provided a model with an improved, higher AUC at 0.83 (Table 2; Figure 1A). The MoM analysis resulted in similar AUC values to ones from a logistic regression analysis (Table 2A and B). All individual and combined biomarker panels provided AUCs that were significantly higher than the null of 0.50 (p < 0.05; Table 2). For example, the data from combined biomarker panel gave an AUC of 0.81 using MoM analysis (Figure 1B).

**Table 2:** **ROC curve area comparison**

***A. From a logistic regression analysis***						
**Biomarker**	**Transformation**	**AUC** ^**a**^	**Standard error**	**95% Wald Confidence Limits**		**Probability** ^**b**^
				**Lower**	**Upper**	
NY-ESO-1	Raw	0.60	0.04	0.52	0.67	0.0102
CEA	Raw	0.79	0.03	0.74	0.85	<0.0001
CA125	Raw	0.70	0.03	0.63	0.77	<0.0001
CYFRA 21-1	Raw	0.69	0.04	0.62	0.76	<0.0001
4 Biomarker Model^c^	Raw	0.83	0.03	0.78	0.88	<0.0001
***B. From a MoM analysis***						
**Biomarker**	**Transformation**	**AUC** ^**a**^	**Standard error**	**95% Confidence Interval**		**Probability** ^**b**^
				**Lower**	**Upper**	
NY-ESO-1	Raw	0.60	0.04	0.52	0.67	0.01007
CEA	Raw	0.79	0.03	0.74	0.85	<0.0001
CA125	Raw	0.70	0.03	0.63	0.77	<0.0001
CYFRA 21-1	Raw	0.69	0.04	0.62	0.76	<0.0001
4 Biomarker Model^c^	Raw	0.81	0.03	0.75	0.86	<0.0001

^**a**^AUC.

^b^Probability of difference to AUC = 0.5.

^c^4 Biomarker model = NY-ESO-1, CEA, CA125, and CYFRA 21-1.

The sensitivity and specificity of individual biomarkers and the combined 4 biomarker panel in the training set were evaluated using both MOM and MLR analysis, which gave very similar results. For example, MOM analysis showed 63%, 42%, 45%, and 47% sensitivity at 80% specificity for CEA, CA125, CYFRA 21–1, and NY-ESO-1, respectively. Although the expressions of each of these 4 biomarkers are elevated in serum of a proportion of patients with LC, they are not sensitive or specific enough to reliably detect asymptomatic patients with LC when considered individually. Combining the tumor antigens (CEA, CA125, and CYFRA 21–1) with the NY-ESO-1 autoantibody marker provides the panel with significantly enhanced sensitivity (73% at 80% fixed specificity) using a cutoff of 6.4. Samples with the aggregate score above this cutoff were considered positive. There was no indeterminate zone in this study. The 4-biomarker panel appears to have good discriminating power to differentiate control patients from LC patients with AUC of 0.81 using MoM method and AUC of 0.83 using MLR analysis (Figure 1A and B).

### Validation set

To further evaluate the diagnostic performance of the 4-biomarker models, the logistic regression parameter estimates and median results for the MoM transformation developed for the training set were applied to predict cancer and control outcomes of the validation data set. Individual biomarker performances in the validation set were comparable with those from the training set. Descriptive statistics for each of the biomarkers are given for the training and validation data sets (Table 3). The combined 4 biomarker panel applied to the validation cohort provided an AUC of 0.82 using the MLR method (p < 0.0001, see Figure 1C). The MoM transformation provided a higher AUC of 0.85 (p < 0.0001, see Figure 1D).

**Table 3:** **Expression levels of biomarkers CEA (ng/ml), CA125 (U/ml), CYFRA 21-1 (ng/ml), and NY-ESO-1 (MFI) for LC cases and matched controls in the training (n=230) and validation (n=150) data sets**

**Biomaker**	**Statistic**	**cancer_train**	**cancer_valid**	**control_train**	**control_valid**
CA125	N	115.00	75.00	115.00	75.00
	Mean	16.76	42.18	9.55	8.47
	StdDev	18.85	135.95	7.71	7.29
	Min	2.19	3.56	0.00	0.00
	Max	170.10	1181.4	41.77	54.96
CEA	N	115.00	75.00	115.00	75.00
	Mean	6.81	9.60	1.35	1.41
	StdDev	13.87	18.01	0.85	1.03
	Min	0.34	0.25	0.04	0.00
	Max	93.37	71.35	6.28	5.96
CYFRA 21-1	N	115.00	75.00	115.00	75.00
	Mean	15.58	4.60	5.93	1.93
	StdDev	55.49	4.34	9.67	1.73
	Min	0.00	0.00	0.00	0.00
	Max	553.85	21.88	89.09	7.17
NY-ESO-1	N	115.00	75.00	115.00	75.00
	Mean	158.17	133.00	49.76	55.68
	StdDev	442.34	290.50	71.12	63.24
	Min	3.37	4.00	3.33	0.00
	Max	3298.3	1890.8	417.70	420.33

The MoM analysis provided a higher AUC for the validation data set than MLR (Figure 1C and D) and, at a specificity of 80%, the MoM 4-biomarker model provided a higher sensitivity as well (77% vs 68%). Holding the training cutoff at 6.4 resulted in an estimate of 71% sensitivity and 88% specificity. Overall, the validation set data support the classification that was determined from the training set and confirmed the ability of the 4-biomarker panel to distinguish early-stage cancers from normal controls**.** All subsequent analysis was performed using the MoM method.

### Diagnostic performance

The validation and training sets were combined to calculate the sensitivity of individual biomarkers at fixed 80% specificity using the MoM method (n = 380). ROC analysis of the combined 4-biomarker panel resulted in 74.2% sensitivity and 80% specificity of NSCLC detection corresponding to a 6.1 cutoff (AUC = 0.83). Applying the classification cutoff of 6.4 from the training set yielded a sensitivity of 72% and a specificity of 83% for the combined set. As before, the individual performances of the four biomarkers showed insufficient sensitivity at 80% specificity (data not shown).

**Figure 2:**
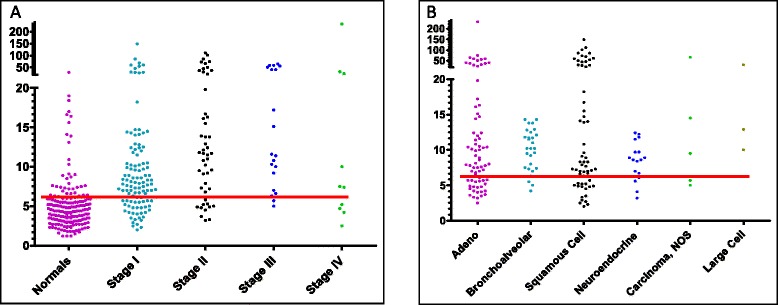
**Distribution of PAULA’s test scores by NSCLC stage (A) and by histological types (B) using MoM model.** The red horizontal lines show the PAULA’s test cut-off of 6.4 derived from training set results.

Performance of the 4-biomarker panel in the combined data set was further characterized through analysis of final PAULA’s scores distribution by tumor stage and tumor histology (Figure 2A and B).

Within the LC group, PAULA’s test scores were grouped according to stages and histological types (Table 4). Early stages (I and II) were detected with less sensitivity and diagnostic accuracy than late stages (71.2% sensitivity and AUC 0.82, p < 0.0001 versus 76.7% sensitivity and AUC 0.87, p < 0.0001). Patients with bronchioalveolar and large cell carcinoma (which included 17 cases of large cell neuroendocrine carcinoma subtype, LCNEC) were detected with the highest sensitivity (85.7% and 75%) and diagnostic accuracy (AUC 0.89 and 0.84, respectively). The most prevalent NSCLC types, adenocarcinoma and squamous cell carcinoma (SCC), demonstrated similar performance in this study (sensitivities 69% and 70%; AUC 0.82 and 0.80, respectively, p < 0.0001) (Table 4). 17 neuroendocrine specimens included in our combined sample set belong to the large cell neuroendocrine carcinoma (LCNEC) which is now recognized as a histologically high-grade NSCLC [49]. Similar to SCLC, LCNEC is a fast-growing type of cancer which represents challenges in early detection and diagnosis. It accounts for approximately 1.6–3.1% of all lung cancer [50]. The LCNEC samples were overrepresented in our LC cases (9.4%). However, they did not affect overall performance of a combined set. When analyzed separately by ROC analysis LCNEC subtype yielded an AUC of 0.84.

**Table 4:** **Performance of PAULA’s test in the combined set (training plus validation) using ROC AUC analysis of 4-biomarker MoM aggregate scores**

	**Number of cases**	**Cutoff**	**AUC**	**P value**	**% sensitivity at 83% specificity**
Early stages (I & II)	160	6.4	0.82	< 0.0001	71.2
Late stages (III & IV)	30	6.4	0.87	< 0.0001	76.7
Total	190				
Adenocarcinoma	73	6.4	0.82	< 0.0001	69.0
Squamous cell carcinoma	60	6.4	0.80	< 0.0001	70.0
Bronchioalveolar carcinoma	28	6.4	0.89	< 0.0001	85.7
Large cell (incl. LCNEC)	20	6.4	0.84	< 0.0001	75.0
Carcinoma, NOS	5	6.4	0.85	0.0069	60.0
Other	4				
Total	190				

The purpose of a PAULA’s LC test is to guide the physician as to the probability of LC and determine the appropriate follow-up procedures based on that probability. Therefore, it is important to know the probability that the test result will give the correct diagnosis. PPV is the proportion of people with a positive test result, either above the MoM or MLR cut off at 80% specificity, who have LC. Assuming a LC prevalence of 2%, a higher proportion of people who have the disease will have a positive test result using the MoM model (7.2%) than the MLR model (6.5%). The NPV is the proportion of people with a negative test result who do not have the disease, which was above 99% for both tests.

### PAULA’s test performance with benign lung diseases and other cancers

To assess the validity of our biomarker panel for discrimination of benign lung conditions from the healthy patient control group, a separate cohort of 81 sera with benign conditions and other cancers was tested using PAULA’s test. Elevated PAULA’s scores (>6.4 cutoff) were found in 9 serum samples (11%). The scores above the 6.4 cutoff were most frequently detected in patients with COPD: 5 out of 9 false-positive cases. PAULA’s score was also elevated in patients with pneumonia (1), asthma (1), inflammation A (1) and in the case of multiple myeloma (1).

Increased expression of CEA and CYFRA 21–1 biomarkers was responsible for the majority of the false positives: 7 out of 9 cases had increased CEA, and 4 out of 9 cases had elevated CYFRA 21–1 expression. NY-ESO-1 autoantibody biomarker was expressed in the serum of a patient with multiple myeloma.

## Discussion

There has been a wealth of recent literature in which the utility of tumor protein-based biomarker or autoantibody panels for use in early LC diagnosis has been evaluated. Historically, the primary impediments to employment of these types of assays in clinical testing have been (1) insufficient specificity, especially using panels with a limited number of biomarkers; and (2) insufficient sensitivity and reproducibility between sample sets.

Our unique approach involves measuring the levels of both of these 2 distinct types of analytes circulating in the blood, tumor antigens and autoantibodies, and using a MoM algorithm to generate a score categorizing the risk that the patient has LC based on the expression of a specific combination of these analytes. This defined combination has the benefit of increasing the sensitivity and specificity of this test as compared to using each analyte type alone.

20/20’s panel is derived from an original 8-biomarker panel that had been previously identified [48]. The markers selected were 3 tumor proteins (CEA, CA125, CYFRA 21–1) supplemented by NY-ESO-1 autoantibodies. Each of the above tumor protein markers has been extensively characterized individually and validated in the scientific literature [51-67]. Overall, the performance of each biomarker included in our study, when analyzed individually, was similar to what has previously been reported in the literature.

CYFRA 21–1 (cytokeratin 19) has been proposed as an independent and sensitive tumor marker for NSCLC since 1993 [51,52]. It has a high diagnostic sensitivity for the detection of NSCLC, especially squamous cell carcinoma (SCC) [19,51-53]. In most cases increased expression levels of CYFRA 21–1 in the blood correlate with the stage of the disease, which can be used for prognostic purposes [54-57]. The prognostic value of serum CYFRA 21–1 levels in NSCLC was estimated in a paper by Pujol et al. [56] where 2063 NSCLC patients data were examined using a meta-analysis of several studies. Results of this analysis showed that an elevated pretreatment CYFRA 21–1 level (>3.6 ng/mL) was an unfavorable prognostic factor in NSCLC. Using 3.3 ng/ml as the cutoff, abnormal CYFRA 21–1 levels were found by Molina et al. in 65.6% of patients with NSCLC [58]. Based on our data, CYFRA 21–1 detected NSCLC with 47.4% sensitivity at 80% fixed specificity and AUC 0.69 (p < 0.0001) when considered alone, which corresponded to a cutoff of 5.1 ng/ml.

Several studies have indicated that CYFRA 21–1 has a better sensitivity for squamous cell carcinoma than other histologies [59]. We also observed higher diagnostic value for CYFRA 21–1 in detecting SCC (AUC 0.71) than adenocarcinoma (AUC 0.63), p <0.0001. However, in our study CYFRA 21–1 expression was most frequently observed in large cell carcinoma (including large cell neuroendocrine carcinoma), resulting in the highest diagnostic accuracy for detecting this histological type (AUC 0.81) compared to other NSCLC subtypes.

CEA is another tumor marker that has been evaluated for its ability to contribute to NSCLC detection [19,60,61] It has relatively high sensitivity for many advanced adenocarcinomas, including lung adenocarcinoma [60]. Several studies have reported that elevated CEA levels indicate poor prognosis in patients with NSCLC, even for those with stage I diagnosis [57,62,63]. Okada et al. [63] measured serum CEA levels in 1,000 NSCLC patients, before and after tumor resection, and found that elevated levels of preoperative CEA correlated with significantly lower 5-year survival compared to patients with normal levels (53.8% versus 75.2%; p < 0.0001). Our data show that CEA has elevated levels (higher than 3 ng/ml) in 46.3% of NSCLC cases (*n* = 190) and 4.7% of normal controls (AUC = 0.77, p < 0.0001). Similar to previous observations, in our study CEA detected adenocarcinoma (using 3 ng/ml cutoff) with a higher sensitivity and diagnostic accuracy than SCC (53.5% versus 40% and AUC 0.8 versus 0.74, respectively). Seventy-five % of the patients with large cell tumors also had elevated CEA levels (>3 ng/ml). This biomarker demonstrated the best sensitivity and diagnostic accuracy, when taken alone, of all the biomarkers evaluated in our study.

The CA125 biomarker has been widely researched as well and mainly used for monitoring ovarian cancer [64]. However, LC can also cause elevated levels of CA125 [19,61,65]. Among LC types, abnormal CA125 serum levels were most often found in adenocarcinoma and large-cell LC [19,59,63]. In our study, CA125 was elevated above normal levels (>35 U/ml) in 18% of NSCLC serums (*n* = 190) and only in 2% of normal controls (*n* = 190). CA125 biomarker sensitivity was the highest in bronchioalveolar carcinoma subtype: 36% of cases had CA125 level higher than 35 U/ml**.** The tumor markers from our panel had varied sensitivities for different histologies, which could aid in distinguishing the type of NSCLC. Additionally, in agreement with the literature reports, the expression of these biomarkers was higher in advanced NSCLC stages as compared to early stages.

Many studies have reported that autoantibodies appear before apparent clinical signs of LC [37-39]. Therefore, they can be detected in serum of patients with the asymptomatic, early stages of cancer. The presence of antibodies to NY-ESO-1 has also been correlated with the progression towards malignancy and worse prognosis in patients with NSCLC [38,64,65]. In this study NY-ESO-1 autoantibodies, when measured individually, had 43% sensitivity at 80% specificity with respect to NSCLC detection. The expression of NY-ESO-1 was much higher in the bronchioalveolar carcinoma subtype, compared to adenocarcinoma and squamous cell carcinoma (82.1%, 42.5%, and 36.7%, respectively). It was not expressed in the neuroendocrine subtype of large cell carcinoma. The sensitivity of early NSCLC stages detection by NY-ESO-1 autoantibodies was 10% higher in comparison with late stage detection (44.7% versus 34.5%, respectively). However, similar to previous observations, no single biomarker analyzed in our study showed sufficient sensitivity for early-stage disease detection when measured individually.

The primary insight provided by the initial work in biomarker panel selection was the realization that even assays that detect panels of several tumor protein markers provide inconsistent results with respect to reliable cancer detection in its early stages. The addition of autoantibody detection to the assay may identify cancer at an early stage due to the fact that autoantibody expression is stage independent. Thus, we combined 3 tumor proteins (CEA, CA125, and CYFRA 21–1) with NY-ESO-1 autoantibodies and were able to improve NSCLC detection sensitivity. The results of ROC analysis using aggregate MoM score demonstrated that our 4-biomarker panel was able to discriminate cases from controls with 72.7% and 82.8% sensitivity at 80% specificity for early- and late-stage NSCLC respectively. NY-ESO-1 autoantibodies in addition to 3 tumor markers resulted in the increase of overall sensitivity of NSCLC detection (from 67.9% for 3 tumor markers combined to 74.2% for 4-biomarker panel), which was mainly due to the increased sensitivity of early-stage detection (from 65.2% to 72.7%, respectively). For LC prevalence in a high-risk population of approximately 2% [68] this performance would result in a PPV of 7.2% and an NPV of 99.4%**.** We believe that the clinical performance of the PAULA’s test, as well as its simple and easy-to-use format, makes it suitable for use as an aid to CT screening.

An interesting observation is that the combination of selected markers provides only a moderate (though important) improvement in assay performance (AUC, sensitivity, specificity) in the current PAULA’s test, as compared to the best individual markers considered alone. This behavior is commonly observed in the development of tumor protein-based assay panels for cancer detection. This indicates that most tumors overlap considerably in the tumor protein markers they express, suggesting that in most cases the assembly of a tumor protein marker panel will be a challenging exercise. Interestingly, there is evidence that autoantibodies are not so redundant, meaning patients positive for one are not often positive for another (from Abbott biomarker selection studies described in Materials and methods, data not shown). This may turn out to be an additional advantage of the mixed tumor protein/autoantibody panel, when additional autoantibodies are added to the present PAULA’s panel.

There are 2 possible scenarios for the placement of an adjunct to CT LC blood test: (1) before diagnostic CT, to predict the risk of LC in order to enrich the population being offered CT scan; and (2) after CT, to improve interpretation of CT images in the setting of suspicious pulmonary nodules.

In this study we investigated whether we could detect early stages of NSCLC using our current 4-biomarker panel. The goal was to identify early tumors in high-risk patients (smokers or former smokers) where disease is curable. Consequently, the majority of cancers in the training and validation sets were retrospective samples from stages I and II. The study’s control population was matched by smoking history, age, and gender and represented people with no history of cancers or other diseases.

We understand the need for studies involving an appropriate control population, such as serum from patients with pulmonary diseases and other cancers, to fully evaluate the current biomarker panel. For this reason we separately tested an additional sample cohort comprised of 81 serum samples from patients with benign diseases (COPD, asthma, bronchitis, pulmonary fibrosis, etc.) and other cancers, including myeloma, breast and colon cancers. We found that PAULA’s test score was above the 6.4 cutoff in 11% of serums from this set. This misclassification was expected due to previous reports in the literature where increased expression of some of these biomarkers was observed in benign pulmonary diseases [69-71] and other cancers. In the benign diseases cohort, 5 out of 8 false positives were COPD cases. Thus, COPD could potentially yield a higher score than normal controls in our test. Previous studies indicate that approximately 20% of heavy smokers develop COPD and the prevalence of this disease is around 50% in patients who had newly diagnosed lung cancer [72]. In addition, it was documented that COPD is an independent risk factor for lung cancer. Therefore, if among false positives for PAULA’s test COPD cases were detected, it should be recognized that this represents the probability that an individual will have lung cancer, and these data should be interpreted cautiously. Elevated PAULA’s scores were also found in patients with 3 other non-neoplastic conditions (pneumonia, asthma, and chronic inflammation), confirming that some inflammatory conditions can modify our biomarkers measurement.

As one of the limitations of the present study, samples with benign pulmonary nodules were not included among the controls. There were 2 primary reasons for this omission: (1) It is difficult and expensive to gain access to serum samples from patients with well characterized pulmonary nodules; and (2) we were not attempting to establish the correlation between positive PAULA’s test and the presence of CT nodules in this study.

The samples used in our study do not represent the expected distribution of cancer types, stages, or the frequency of benign diseases that one would see in the screening population of high risk smokers. This is a common problem with the usage of archived sample sets in validation studies such as this, and slightly altered performance must be expected when implementing this test in the general population. There is no reason to expect that these results are not predictive of real-life performance when PAULA’s test is implemented as a clinical test. Nevertheless, a prospective study will be required to further validate the utility of PAULA’s assay for clinical use. We are currently testing clinical samples from the high-risk population in our CLIA lab. The follow-up information from the positives by the PAULA’s test cases is being collected and will help to estimate the test’s clinical utility with an intended use population.

To further increase the sensitivity and specificity of this panel in the detection of early LC stages, the addition of other biomarkers (autoantibodies and tumor proteins) to our present panel is currently in development by our laboratory. The current biomarker panel will be refined as follow-up data accrue. We anticipate that future additions of new biomarkers will result in a better clinical performance.

## Conclusion

In conclusion, we have presented a validation study of 20/20’s PAULA’s test that effectively distinguished NSCLC cases from normal patients and patients with benign pulmonary diseases. Based on these and previous data we believe that the performance of the PAULA’s test makes it suitable for use as an aid to LDCT screening. The complementary use of an annual blood-based biomarker test to establish a suspicion of cancer risk before patients would be subjected to CT holds great promise for the early detection of LC, leading to a reduction in the disease-specific mortality rate. There remains a great deal of controversy regarding LDCT screening at many levels, including long-term radiation exposure, the high number of false positives, lack of standardization of the LDCT procedure, and analysis outside of a clinical trial environment. For physicians, a serum test that could be used as a screening tool to identify patients with suspected asymptomatic LC and guide them to seek secondary (diagnostic) CT screening is urgently needed. As noted, our data suggest this would be a productive strategy. From a healthcare perspective, such pre-enrichment of cancer risk prior to CT would in effect avoid a portion of false positive CT results and consequent workups, and would enhance the cost effectiveness of screening.
